
JOURNAL INFORMATION
==============================
NLM Title Abbreviation: Biospektrum (Heidelb)
Journal ID: Biospektrum (Heidelb)
NLM Journal ID: 9615685

Biospektrum

ISSN: 0947-0867
EISSN: 1868-6249

ARTICLE INFORMATION
==============================
PMCID: PMC10101536
PMID: 37073323
DOI: 10.1007/s12268-023-1907-x
Article ID: 1907
Article version: 1
Subjects: Wissenschaft · Special: Molekulare Medizin & Virologie

Strukturierte RNAs in Viren

Ochsenreiter Roman 1
Wolfinger Michael T. michael.wolfinger@univie.ac.at https://michaelwolfinger.com
1 2 3
1 grid.10420.37 0000 0001 2286 1424 Institut für Theoretische Chemie, Universität Wien, Wien, Österreich
2 grid.10420.37 0000 0001 2286 1424 Arbeitsgruppe Bioinformatik und Computational Biology, Fakultät für Informatik, Universität Wien, Währinger Strasse 29, A-1090 Wien, Österreich
3 RNA Forecast E. U., Wien, Österreich
Electronic publication date: 2023 Apr 13
Print publication date: 2023
Volume: 29
Issue: 2
First page: 156
Last page: 158
Copyright: © Die Autoren 2023
License: Open Access: Dieser Artikel wird unter der Creative Commons Namensnennung 4.0 International Lizenz veröffentlicht, welche die Nutzung, Vervielfältigung, Bearbeitung, Verbreitung und Wiedergabe in jeglichem Medium und Format erlaubt, sofern Sie den/die ursprünglichen Autor(en) und die Quelle ordnungsgemäß nennen, einen Link zur Creative Commons Lizenz beifügen und angeben, ob Änderungen vorgenommen wurden. Die in diesem Artikel enthaltenen Bilder und sonstiges Drittmaterial unterliegen ebenfalls der genannten Creative Commons Lizenz, sofern sich aus der Abbildungslegende nichts anderes ergibt. Sofern das betreffende Material nicht unter der genannten Creative Commons Lizenz steht und die betreffende Handlung nicht nach gesetzlichen Vorschriften erlaubt ist, ist für die oben aufgeführten Weiterverwendungen des Materials die Einwilligung des jeweiligen Rechteinhabers einzuholen. Weitere Details zur Lizenz entnehmen Sie bitte der Lizenzinformation auf http://creativecommons.org/licenses/by/4.0/deed.de.
License URL: https://creativecommons.org/licenses/by/4.0/

issue-copyright-statement: © Springer-Verlag GmbH Deutschland, ein Teil von Springer Nature 2023

==============================
Evolutionarily conserved RNAs in untranslated regions are key regulators of the viral life cycle. Exoribonuclease-resistant RNAs (xrRNAs) are particularly interesting examples of structurally conserved elements because they actively dysregulate the messenger RNA (mRNA) degradation machinery of host cells, thereby mediating viral pathogenicity. We review the principles of RNA structure conservation in viruses and discuss potential applications of xrRNAs in synthetic biology and future mRNA vaccines.

▲ **Abb. 1:** RNA und ihre Fähigkeit zur Bildung von Struktur. **A**, Wechselwirkungen zwischen den Nukleotidbasen. **B**, schematische Abbildung einer Nukleotidkette und möglicher Basenpaare, die nicht unmittelbar benachbarte Nukleotide in räumliche Nähe zueinander bringen. **C**, strukturierte RNAs in viralen Genomen. **D**, detaillierte Sekundärstruktur und **E**, Tertiärstruktur einer funktionalen RNA.

■ Flaviviren (Gattung: *Flavivirus*) umfassen rund 150 bekannte Viren, darunter wichtige Humanpathogene, wie Dengue-, Gelbfieber- oder FSME-Viren (*tick-borne encephalitis virus*, TBEV). Flaviviren zirkulieren typischerweise zwischen Insekten, wie Stechmücken oder Zecken, als Überträger (Vektoren) und Wirbeltieren als Wirt. Obwohl der Mensch für die wenigsten Flaviviren ein geeigneter Wirt ist, sind Infektionen häufig und führen jährlich zu Millionen von Erkrankungen, die Gesundheitssysteme global vor eine Herausforderung stellen.

## Flaviviren: Viren mit einem RNA-Genom

Flaviviren gehören zu der großen Gruppe der RNA-Viren. Im Unterschied zu vielen anderen Viren, Bakterien oder höheren Lebewesen, die DNA als Informationsträger verwenden, ist ein RNA-Genom nicht ausschließlich Träger der Erbinformation, sondern übernimmt gleichzeitig auch regulative Funktionen. Dazu gehören u. a. die Steuerung der viralen Replikation, Proteinsynthese oder die Umgehung der Immunantwort des Wirts.

## RNA: dynamisch und stark strukturiert

Eine Besonderheit von RNA ist ihre Fähigkeit, komplexe Strukturen auszubilden, indem einzelnen Bausteine (Nukleotidbasen) durch Basenpaarungen miteinander wechselwirken (**Abb. 1**). Die Gesamtheit der Basenpaarungen wird als Faltung oder Sekundärstruktur eines RNA-Moleküls bezeichnet. Während Sekundärstrukturen keine exakte Beschreibung der dreidimensionalen Struktur darstellen, bilden sie dennoch eine hinreichend genaue Näherung, um die biologische Funktion zu erklären und sind daher von großer Bedeutung für die Forschung. In diesem Zusammenhang ist es interessant sich zu verdeutlichen, dass die Abfolge der Nukleotidbasen letztendlich bestimmt, welche Sekundärstrukturen ausgebildet werden können. Beim Vergleich der RNA-Genome (Primärsequenzen) evolutionär verwandter Flaviviren, wie z. B. Dengue- und Zika-Viren, fällt auf, dass sich diese stark unterscheiden. Andererseits weisen die davon ausgebildeten RNA-Strukturelemente eine hohe Ähnlich-

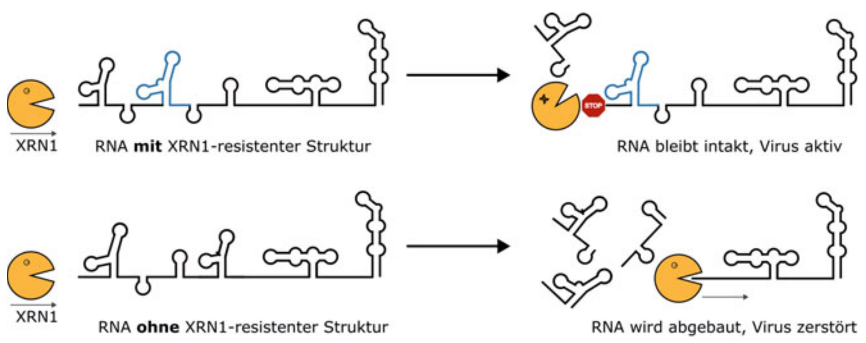

▲ **Abb. 2:** RNA-Strukturelemente von Flaviviren verhindern den Abbau der viralen RNA durch das wirtseigene Protein XRN1. Die RNA-Struktur agiert dabei als Blockade und stellt sicher, dass XRN1 keine weitere RNA mehr abbaut, sobald es dem Strukturelement begegnet.

keit auf. Dieser Effekt ist als RNA-Struktur-Konservierung bekannt und lässt sich auf der Ebene der Sekundärstrukturen effizient beschreiben [1, 2].

### Flaviviren besitzen evolutionär konservierte RNA-Struktur

Das RNA-Genom von Flaviviren ist an beiden Enden von hochstrukturierten Bereichen flankiert, die nicht für Proteine codieren. Am Ende des Virus-Genoms, innerhalb der 3'-untranslatierten Region (3'UTR), finden sich zahlreiche strukturierte RNA-Elemente, die spezifische biologische Funktionen übernehmen. Vergleiche verwandter Flaviviren haben gezeigt, dass strukturell und funktionell ähnliche RNAs in diesem Bereich des viralen Genoms häufig vorkommen. Es ist daher naheliegend, dass es evolutionären Druck auf die Erhaltung bestimmter RNA-Strukturen gibt, wohingegen dieser Effekt auf der Ebene der Primärsequenzen wenig ausgeprägt ist. Konkret wirkt dieser evolutionäre Druck auf die Erhaltung von Basenpaaren. Wenn zum z. B. in einem für die Struktur wichtigen A–U-Basenpaar eine A→C-Mutation auftritt, lastet in Folge dessen evolutionärer Druck auf dem Basenpaarungspartner U und begünstigt eine weitere Mutation U→G, die das Basenpaar wiederherstellt. Dieses Phänomen ist als kompensatorische Mutation bekannt und von eminenter Bedeutung für die Identifikation von konservierten RNA-Strukturen.

### xrRNAs: RNA-Struktur als virale Überlebensstrategie

Ein in Flaviviren gut untersuchtes Beispiel für evolutionäre Konservierung funktioneller RNA-Elemente ist die Familie der Exoribonuklease-resistenten RNAs (xrRNAs), die es den Viren ermöglicht, den regulären mRNA-Abbau in infizierten Zellen aktiv zu unterbinden (**Abb. 2**). In diesem Zusammenhang spielt die wirtseigene 5'-3'-Exonuklease XRN1 [3] eine besondere Rolle: Flaviviren haben die bemerkenswerte Strategie entwickelt, einen partiellen Abbau des eigenen Genoms zu tolerieren, mit dem Ziel, die Exonuklease an den hochkonservierten xrRNA-Elementen in der 3'UTR irreversibel zu blockieren und den Abbau dahinterliegender RNA-Regionen zu verhindern. Derart blockierte Exonukleasen stehen der Zelle de facto nicht mehr zur Verfügung, wodurch der Abbau weiterer Viruspartikel erschwert wird.

### Umgehung des Immunsystems durch virale RNAs

Als unmittelbare Konsequenz dieser Blockade von Exonukleasen entsteht eine Ansammlung von langen nicht codierenden viralen RNAs (*long non-coding RNA*, lncRNA) in infizierten Zellen. Diese subgenomischen flaviviralen RNAs (sfRNAs) sind stabile Abbauprodukte, die aus unvollständigem Abbau der viralen RNA durch die Exoribonuklease-Tätigkeit des Wirts entstehen [4]. sfRNAs beeinträchtigen essenzielle zelluläre Prozesse, wie beispielsweise den Umsatz von mRNAs oder die antivirale Typ-I-Interferon-Antwort in Vertebraten mit dem Ziel, eine virale Infektion voranzutreiben [5]. Quantitativer Schutz gegen Abbau durch Exonukleasen, der zur Bildung bzw. Akkumulation von sfRNAs führt, dürfte eine universelle Eigenschaft aller Flaviviren sein.

### Flaviviren als evolutionärer Hotspot für Strukturevolution xrRNAs kommen nicht nur in praktisch allen bekannten Flaviviren vor, sondern weisen auch subtile, wenngleich deutlich unterscheidbare strukturelle Alleinstellungsmerkmale auf. Wir konnten mit bioinformatischen Methoden zur RNA-Struktur-vorhersage [6] und Homologiesuche [7]

Hier steht eine Anzeige.

Springer

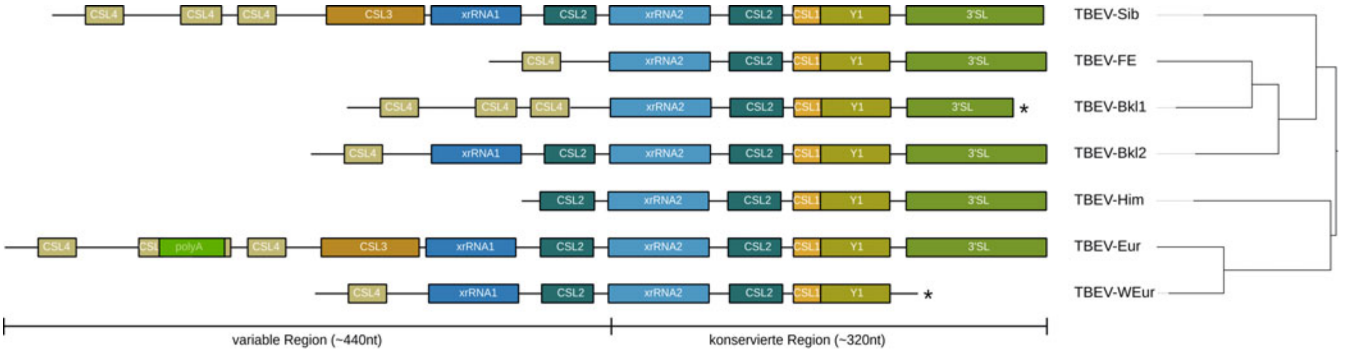

▲ **Abb. 3:** Vergleich der 3’UTRs verschiedener Subtypen des FSME-Virus. Der phylogenetische Baum rechts zeigt die Verwandtschaft der Subtypen an. Die 3’UTRs weisen nicht nur unterschiedliche Längen auf, sondern auch eine abweichende Anordnung funktionaler RNA-Elemente. Strukturell homologe Elemente sind farblich gekennzeichnet. Unvollständige Bereiche sind mit einem Stern gekennzeichnet. Abbildung angepasst von [8].

zeigen, dass xrRNA-Elemente in Flaviviren typischerweise in zwei (in Extremfällen sogar bis zu fünf) hintereinander liegenden Kopien vorkommen [1]. Dabei scheint es sich um eine Backupstrategie der Viren zu handeln, die sicherstellt, dass auf jeden Fall sfRNAs gebildet werden, selbst wenn ein xrRNA-Element durch XRN1 abgebaut werden sollte. Es gibt aber auch Beispiele von Flaviviren, die nur eine xrRNA in ihrer 3’UTR besitzen, wie der Dengue-Virus Serotyp IV. Dessen einzelne xrRNA ist homolog zur zweiten xrRNA in den Dengue-Serotypen I–III, was darauf schließen lässt dass Dengue-Serotyp IV eine xrRNA im Lauf der Evolution verloren hat [2]. Ein anderes interessantes Beispiel sind die FSME-Viren, von denen bis dato sieben Subtypen beschrieben sind. Diese Subtypen unterscheiden sich nicht nur in ihrer Pathogenität, sondern weisen allesamt unterschiedliche 3’UTR-Architekturen auf (**Abb. 3**), die sich aus der Kombination von insgesamt acht evolutionär konservierten RNA-Elementen ergeben [8].

## Strukturierte RNAs und mRNA-Impfstoffe

Die SARS-CoV-2-Pandemie hat gezeigt, dass RNA-Viren in der Lage sind, die globale Ordnung nachhaltig zu beeinträchtigen. Gleichzeitig hat sich Dank der Effektivität der mRNA-Impfstoffe eine Technologie entwickelt, die der Schlüssel zur Bekämpfung zukünftiger Pandemien ist. Obwohl bei der Entwicklung der aktuellen mRNA-Impfstoffe nicht auf RNA-Struktur geachtet wurde [9], ist hier dennoch ein enormes Potenzial für die kommende Generation von mRNA-Impfstoffen vorhanden. Gegenstand unserer aktuellen Forschung ist es, künstliche xrRNAs zu designen, die als molekulare Schalter den Abbau von mRNAs ermöglichen oder verhindern. Diese Technologie aus dem Bereich der synthetischen Biologie zur Regulation der Stabilität von RNA-Molekülen wird uns in weiterer Folge erlauben, die Geschwindigkeit, mit der z. B. ein mRNA-Impfstoff abgebaut wird, genau zu kontrollieren. Durch eine genaue Kontrolle der Halbwertszeit von mRNA-Molekülen besteht das Potenzial, die Proteinexpression zu verlängern und dadurch die Effektivität von RNA-basierten Therapien zu erhöhen. ■


Funding note: Open Access funding provided by University of Vienna.

Michael T. Wolfinger 1995–2001 Chemiestudium. 2004 Promotion. 2005–2011 Postdoktorand. 2012–2018 Leitung Bioinformatik für mehrere Spezialforschungsbereiche. Seit 2019 Arbeitsgruppe Bioinformatik und Computational Biology, Universität Wien. Seit 2022 Consultant für RNA Computational Biology. 2023 Gründung RNA Forecast e. U. mit Fokus RNA als small molecule drug target und strukturbasiertes Design für mRNA-Impfstoffe.

Roman Ochsenreiter 2009–2015 Studium der Molekularen Biologie und Bioinformatik. 2015–2019 Doktorand am Institut für Theoretische Chemie, Universität Wien. 2019–2023 Leitung Bioinformatik bei Biotech-Unternehmen. Seit 2023 Consultant für Bioinformatik mit Schwerpunkt RNA-Bioinformatik, Humangenetik und Computational Biology.


Literatur

[1] Ochsenreiter R Hofacker I L Wolfinger MT Functional RNA Structures in the 3’UTR of Tick-Borne, Insect-Specific and No-Known-Vector Flaviviruses Viruses 2019 11 298 10.3390/v11030298 30909641 PMC6466055
[2] Wolfinger MT Ochsenreiter R Hofacker IL Frishman D Marz M Functional RNA Structures in the 3’UTR of Mosquito-Borne Flaviviruses Virus Bioinformatics 2021 Chapman Hall/CRC Press 65 100
[3] Jones CI Zabolotskaya MV Newbury SF The 5’-3’ Exoribonuclease Xrn1/Pacman and Its Functions in Cellular Processes and Development Wiley Interdiscip Rev RNA 2012 3 455 468 10.1002/wrna.1109 22383165
[4] Pijlman GP Funk A Kondratieva N A Highly Structured, Nuclease-Resistant, Noncoding RNA Produced by Flaviviruses Is Required for Pathogenicity Cell Host Microbe 2008 4 579 591 10.1016/j.chom.2008.10.007 19064258
[5] Slonchak A Khromykh AA Subgenomic Flaviviral RNAs: What Do We Know After the First Decade of Research Antivir Res 2018 159 13 25 10.1016/j.antiviral.2018.09.006 30217649
[6] Lorenz R Bernhart S H Höner Zu Siederdissen C ViennaRNA Package 2.0 Algorithm Mol Biol 2011 6 26 10.1186/1748-7188-6-26 PMC3319429 22115189
[7] Nawrocki EP Eddy SR Infernal 1.1: 100-Fold Faster RNA Homology Searches Bioinformatics 2013 29 2933 2935 10.1093/bioinformatics/btt509 24008419 PMC3810854
[8] Kutschera LS Wolfinger MT Evolutionary Traits of Tick-Borne Encephalitis Virus: Pervasive RNA Structure Conservation and Molecular Epidemiology Virus Evol 2022 8 veac051 10.1093/ve/veac051 35822110 PMC9272599
[9] Xia X Detailed Dissection and Critical Evaluation of the Pfizer/BioNTech and Moderna mRNA Vaccines Vaccines 2021 9 734 10.3390/vaccines9070734 34358150 PMC8310186
